# Prognostic value of integrated morphofunctional imaging methods in inoperable intrahepatic cholangiocarcinoma

**DOI:** 10.3389/fmed.2023.1204717

**Published:** 2023-07-06

**Authors:** Cristina Nanni, Cristina Mosconi, Valentino Dragonetti, Massimo Barakat, Nicola Fraccascia, Maria Adriana Cocozza, Stefano Brocchi, Andrea Palloni, Alexandro Paccapelo, Giovanni Brandi, Stefano Fanti

**Affiliations:** ^1^Nuclear Medicine Unit, IRCCS Azienda Ospedaliero-Universitaria di Bologna, Bologna, Italy; ^2^Radiology Unit, IRCCS Azienda Ospedaliero-Universitaria di Bologna, Bologna, Italy; ^3^Nuclear Medicine, Alma Mater Studiorum, University of Bologna, Bologna, Italy; ^4^Medical Oncology, IRCCS Azienda Ospedaliero-Universitaria di Bologna, Bologna, Italy; ^5^Department of Internal Medicine and Surgery, Alma Mater Studiorum, University of Bologna, Bologna, Italy

**Keywords:** intra-hepatic cholangiocarcinoma, FDG PET/CT, ceCT, prognostic value, TARE

## Abstract

**Introduction and aim:**

Intrahepatic cholangiocarcinoma (iCCA) is a disease characterized by rarity, heterogeneity, and high mortality, where surgical resection is often not possible. Nowadays, due to the recent introduction of new therapeutic options such as trans-arterial radioembolization (TARE), it is increasingly important to define the role of morphofunctional imaging methods for the prognostic stratification of patients affected by iCCA. The aim of the study was to verify the prognostic value of morphofunctional imaging methods at the baseline in patients with inoperable iCCA.

**Methods:**

In total, 45 patients with iCCA were sent to our center between January 2016 and March 2021 for being evaluated to be treated with TARE. All of them underwent both [18F]-FDG-PET/CT and contrast-enhanced CT (ceCT) in a single procedure and were included in our study. The inclusion criteria were as follows: a diagnosis of inoperable iCCA; both [18F]-FDG-PET/CT and ceCT scans; and washout from therapy for at least 2 months before baseline [18F]-FDG-PET/CT and ceCT scans. Both clinical and laboratory data and baseline imaging data (ceCT and [18F]-FDG-PET/CT) were collected. In particular, regarding clinical and laboratory data, we collected overall survival (OS), gender, age, prior therapies, liver function indices, and tumor markers. Regarding ceCT, we collected TNM staging, lesion diameter, volume, vascularization, and presence of intravascular necrosis. Regarding [18F]-FDG-PET/CT, we collected TNM staging, Standard-Uptake-Value max (SUVmax), Metabolic-Tumor-Volume (MTV), and Total-Lesion-Glycolysis (TLG=MTV^*^lesions SUVmean). Philips-Vue-PACS software was used, setting hepatic SUVmean as TLG threshold.

**Results:**

A statistically significant correlation was found between some examined parameters at morphofunctional investigations at the baseline and OS. [18F]-FDG-PET/CT parameters statistically correlated with OS were the stage of disease greater than M0 (*p* = 0.037), major lesion SUVmax (*p* = 0.010), MTV (*p* ≤ 0.001), and TLG (*p* < 0.001). Other parameters at ceCT correlated with OS were the stage of disease greater than T2 (*p* = 0.038), maximum lesion diameter (*p* = 0.07), volume of the major lesion (*p* = 0.016), and total volume of lesions (*p* = 0. 009). Biochemical parameters correlated with OS were gamma glutamyl transferase (GGT, *p* = 0.014), alkaline phosphatase (ALP, *p* = 0.019), carcinoembryonic antigen (CEA, *p* = 0.004), and carbohydrate antigen 19-9 (CA 19-9, *p* < 0.001). From the parameters estimated by the multivariate model, we derived a four-variable score for OS combining nodal involvement and SUVmax at [18F]-FDG-PET/CT, GGT, and CA 19-9 levels.

**Conclusion:**

Considering our data, performing integrated pre-therapy imaging is critical for the prognostic stratification of patients with iCCA.

## 1. Introduction

Cholangiocarcinoma (CCA) is the most common biliary malignancy and the second most common hepatic malignancy after hepatocarcinoma (1, 2), with more than 95% of CCA are ductal adenocarcinomas that arise from the epithelium of the biliary tract (3). Several risk factors, such as parasitic infections, toxins, hepatitis B and C, and primary sclerosis cholangitis (PSC) are correlated with the risk of developing CCA (3).

CCA are divided into three types, according to their anatomical localization, such as intrahepatic cholangiocarcinoma (iCCA), perihilar cholangiocarcinoma (pCCA, also known as Klatskin tumor), and extrahepatic cholangiocarcinoma (4). iCCA is the least common variant of CCA, representing 10–20% of all CCA diagnosed, but several studies globally reported increased rates of iCCA in the last few decades (5–8). Given the prevalence of paucisymptomatic clinical figures and the lack of any screening programs, up to 70% of patients are diagnosed with locally advanced or metastatic disease, precluding surgical intervention, which nowadays represents the only available treatment (9, 10), and, outside curative option, iCCA has a high mortality rate, with only 5–10% of patients with unresectable disease alive 5 years after initial diagnosis (3), with the combination of gemcitabine/cisplatin chemotherapy and immunotherapy, considered as the new first-line treatment option.

Given the current treatment limitations, there is a need for the implementation of new therapy options, such as TACE and TARE, that nowadays are commonly used only for palliative purposes. To do so, it is important to evaluate the role of multimodality imaging, considering its importance not only for diagnosis and staging but also for the correct management of patients with iCCA.

Among the main imaging methods for iCCA, there is ceCT, which is mainly used for diagnosis and staging, with reported sensitivity and specificity values in the literature of nearly 78 and 80%, respectively (11, 12). In particular, it is characterized by the detection of lesions with irregular margins with early peripheral enhancement and late central enhancement. Different studies have shown how ceCT can better assess tumor infiltration of perilesional vascular structures (13), as well as a high capacity to define the resectability of such lesions, with a negative predictive value of 85–100% (14). The diagnostic accuracy of ceCT in the evaluation of lymph node involvement is approximately 77% (15). ceCT can also diagnose distant metastases, frequently involving the peritoneum, lungs, and bone (16).

Another imaging method used in the diagnosis of iCCA is magnetic resonance imaging (MRI), with sensitivity and specificity for small lesions reported in the literature of nearly 77 and 96%, respectively (17).

[18F]-FDG-PET/CT is currently used mainly in the detection of distant metastases, rather than for primary lesions assessment, with a recent meta-analysis showing a sensitivity and specificity of 95 and 93%, respectively (18), which is higher than ceCT for this indication (19, 20).

Some authors demonstrated how some semi-quantitative parameters of [18F]-FDG-PET/CT, such as SUVmax, may be related to the risk of recurrence and OS (21), as well as how additional parameters such as MTV and TLG are predictive of 5-year survival rates (22).

Given this, the aim of our study was to evaluate the impact of baseline [18F]-FDG-PET/CT in the prognostic stratification of patients with inoperable iCCA who reached our center for being evaluated for TARE.

## 2. Materials and methods

The study conforms to the ethics guidelines of the Declaration of Helsinki, and data collection and analyses had already previously been approved by our Institutional Review Board and ethical committee of S. Orsola Hospital (193/2021/Oss/AOUBo). All patients provided informed written consent for the TARE and for the processing of personal data according to the Italian Data Protection Authority law (legislative decree n. 196 of 2003 and n. 101 of 2018).

A total of 45 patients were sent to our center by other centers for being evaluated for an eventual TARE between January 2016 and March 2021. All of them had a diagnosis of inoperable iCCA and were out of therapies for at least 2 months. All the enrolled patients underwent a combined imaging procedure including a [18F]-FDG-PET/CT and ceCT (GE, Discovery MI DR or GE, Discovery D710 equipped with a 64 slices CT). Patients for whom TARE was not indicated underwent different systemic treatments.

Patients were eligible for this treatment if they met the following criteria: (1) histologically proven ICC; (2) unresectable naive tumor or disease relapse/persistence after various treatments, including liver resection; (3) Eastern Cooperation Oncology Group (ECOG) performance status of 0–2; (4) adequate liver function with bilirubin < 2.0 mg/dL; (5) granulocyte count ≥1.5 × 109/L; (6) platelet count ≥50 × 109/L, and (7) amenability to visceral angiography. The exclusion criteria included (1) flow to the gastrointestinal tract not correctable by coil embolization or (2) estimated lung exposition to a radiation dose >30 Gray (Gy).

### 2.1. [18F]-FDG-PET/CT images

[18F]-FDG-PET/CT scans were performed as per the standard procedure suggested by EANM guidelines (23).

Briefly, patients were not allowed to consume any food or sugar for at least 6 h prior to the start of the [18F]-FDG-PET/CT study. The injected dose was 2–3 MBq/Kg. The uptake time was 60 ± 10 min. Pain medications were allowed but corticosteroids were forbidden. The field of view must include the skull up to the mid-thigh. Acquisition time was 2 min/bed position. Low-dose CT was acquired at 120 kV and 80 mA. Iterative reconstruction (3D ordered subsets expectation maximization with 2 iterations, 20 subsets, followed by smoothing with a 6-mm 3D Gaussian kernel) and CT-based attenuation, scatter, random coincidence events, and system dead-time iterative correction of emission data can be applied to obtain high-quality PET images (24).

### 2.2. Computed tomography images

ceCT was performed immediately after the PET study. A solution of 71.44% w/v of iomeprol equivalent to 35% iodine or 350 mg iodine/ml (Iomeron 350) was used as IV contrast media and was administrated in a dosage corresponding to 100–150 ml depending on patient weight.

All the patients underwent a multiphase-modulated CT with the following scheme:

1. thorax, abdomen, and pelvis without i.v. contrast agent
2. superior abdomen in an early arterial phase
3. superior abdomen in a late arterial phase
4. thorax, abdomen, and pelvis in a venous phase
5. abdomen and pelvis in a late phase at 4 min since the contrast media injection.

After infusion of the contrast agent at a rate of 3–4 mL/s, the early hepatic arterial phase was determined using a region of interest (ROI) placement in the abdominal aorta at the level of the celiac artery, and the CT scan was initiated when achieving a peak of at least 150 Hounsfield units (HU) within the ROI; the late arterial phase, venous phase (70–80 s), and a late phase (4 min) were then acquired.

CT images were reconstructed as follows:

- HRCT on the thorax with a lung filter
- soft tissue filter and 2.5 mm slice thickness for the other series.

**Figure 1:**
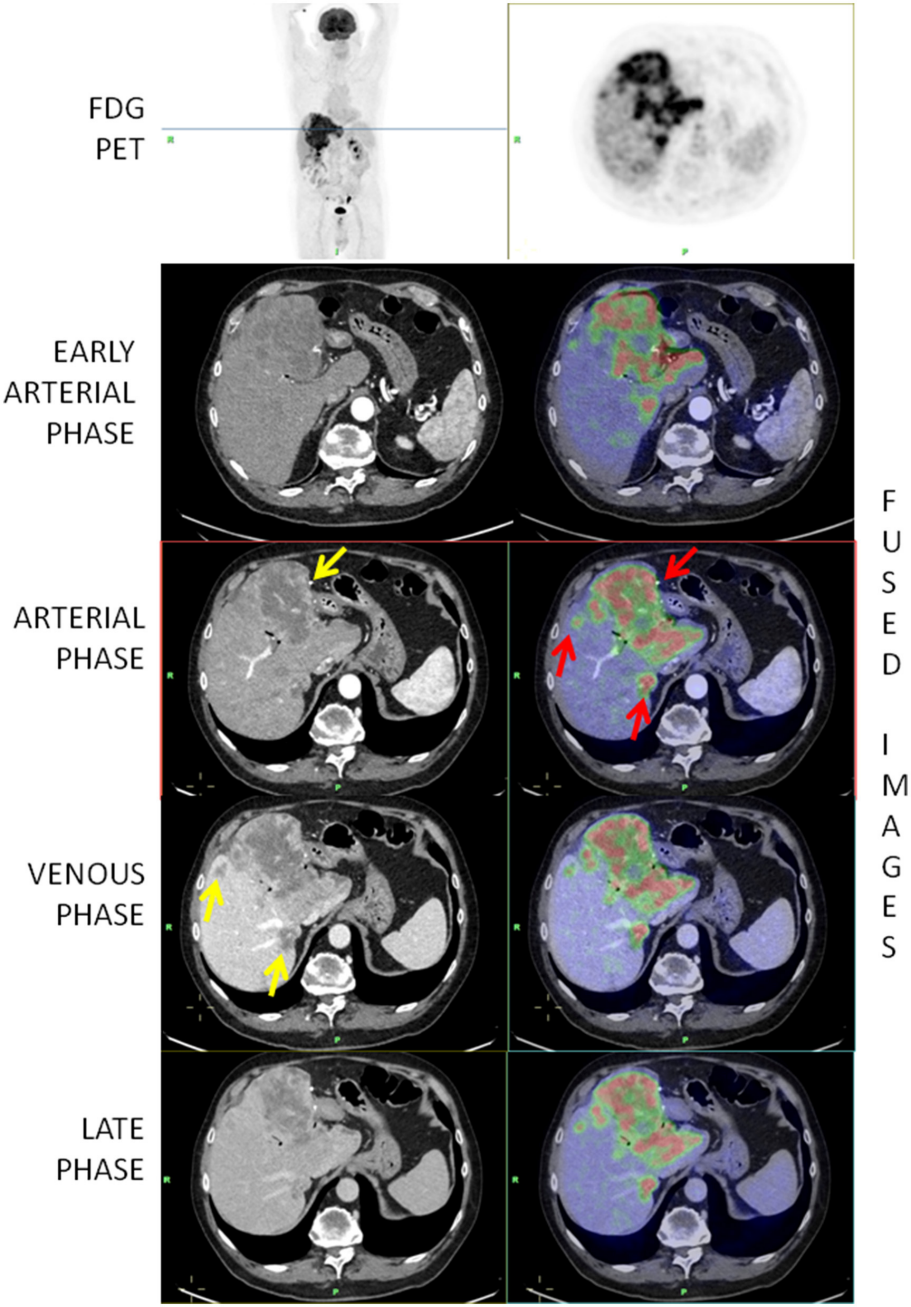
[^18^F]-FDG-PET/ceCT showing a highly metabolic primary iCCA and two hepatic secondary lesions (red arrows). The yellow arrow on the arterial phase shows a peripheral vascularization while the arrows on the venous phase show that secondary lesions are better depicted in the venous phase.

The volume analysis was performed using Philips IntelliSpace portal^®^. The evaluation of intrahepatic vascular and biliary anatomy is fundamental to obtain an accurate liver volume estimation. An expert radiologist (more than 10 years of experience) manually traced the contour of the major lesion and the contour of the total volume of disease, on every slice of portal venous phase CT. Subsequently, the software provides to calculate the volume of the region of interest segmented (Figure 1).

TARE procedure: When indicated, radioembolization therapy was carried out according to a technique previously described in detail elsewhere (25, 26).

Chemotherapy treatment: Patients with residual or intra/extrahepatic progressive disease were treated with systemic chemotherapy according to age, performance status, and liver function. The combination of cisplatin and gemcitabine was the preferred first-line treatment option in patients with good performance status (ECOG PS 0-1) and preserved liver function, while gemcitabine monotherapy was administered in unfit and/or elderly patients.

### 2.3. Staging and follow-up

ceCT data considered were TNM staging, lesion vascularization, presence of intralesional necrosis, maximum lesion transversal diameter (expressed in millimeters), major lesion volume, and total volume of disease (both expressed in square centimeters).

[18F]-FDG-PET/CT parameters evaluated were TNM staging, SUVmax, MTV, and TLG. In order to collect semi-quantitative data, all [18F]-FDG-PET/CT scans were retrospectively re-evaluated, using Philips-Vue-PACS software and setting hepatic SUVmean as TLG threshold.

After treatment, the patients were regularly evaluated at 1 and 3 months, and thereafter at 3-month intervals. At each visit, clinical data were recorded, and CT or magnetic resonance imaging (MRI) was performed. The following blood tests data were also collected: aspartate aminotransferase (AST), alanine transaminase (ALT), GGT, ALP, total bilirubin, CEA, CA 19-9, and alpha fetoprotein (AFP). All clinical, laboratory, and imaging data were prospectively collected.

OS data, with a median follow-up of 47.9 months, were also evaluated and correlated with the above-mentioned variables.

### 2.4. Statistical analysis

Data were reported as mean ± standard deviation or frequencies. The Kolmogorov–Smirnov test with Lilliefors correction was used to assess the distributional normality of the variables. Survival data were estimated and represented graphically by the Kaplan–Meier method. For survival analyses, hazard ratios with their 95% confidence intervals were estimated by univariate and multivariate Cox regression models. The forward stepwise choice procedure based on the likelihood ratio with an input *p*-value of 0.050 and an output *p*-value of 0.100 was used to choose variables in the multivariate models. A score was calculated with parameters estimated from the multivariate model. Best cutoffs were estimated by maximizing the Youden index and evaluating any local maxima for OS at 12 months. Two-tailed tests with a *p*-value of < 0.05 were considered to be statistically significant. All analyses were performed with IBM SPSS 27.0 software (SPSS Inc., Armonk, NY, USA).

From the parameters estimated by the multivariate analysis, we derived a score for OS combining [18F]-FDG-PET/CT, ceCT, and clinical data. The score was therefore calculated according to the following formula: N-PET-stage^*^1.834 + log10(GGT(UL)) ^*^ 0.969 + log10(CA 19-9(Uml) ^*^ 0.714 + SUVmax ^*^ 0.087. We found two local maxima correlated to OS at 12 months, so we grouped the score into three categories considering group 1 for results < 3.5, group 2 between 3.5 and 4.7, and group 3 for values >4.73.

## 3. Results

### 3.1. Patient characteristics

Of the 45 patients enrolled, 25 were men (55.6%) and 20 were women (44.4%), with an average age of 62.9 years (range 29–86y). A total of 31 patients (68.9%) had undergone prior therapies: 11 surgery, 6 local therapies, and 14 chemoembolization.

Information regarding therapy following multidisciplinary imaging methods was available for 21 patients, of whom the indication for TARE was confirmed in 38% of cases, while the others were referred to other systemic treatment approaches.

At ceCT, five patients were categorized as T1 (11.1%), 19 as T2 (42.2%) and 21 as T3 (46.7%); 24 as N0 (53.3%) and 21 as N1 (46.7%); 20 as M0 (44.4%) and 25 as M1 (55.6%).

In the vast majority of cases, the vascularization of the lesions was peripheral (43; 95.6%) (Figure 1), whereas in only one case, the lesions were hypodense and in one case, it was hyperdense. Intralesional necrosis was present in 35 cases (77.8%) and absent in 10 (22.2%).

The mean maximum transverse diameter of the largest lesion was 76.2 mm. The mean of the largest lesion volume was 321.7 cm^3^, and the mean of the total lesion volume was 414.9 cm^3^.

At [18F]-FDG-PET/CT, two patients showed no hypermetabolic primary lesion and were therefore categorized as T0 (4.4%), 13 as T1 (28.9%), 19 as T2 (42.2%), and 11 as T3 (24.4%); 41 as N0 (91.9%) and 4 as N1 (8.9%); 24 as M0 (53.3%), and 21 as M1 (46.7%).

The mean SUVmax was 12.8. The mean MTV was 479.0 cm^3^, with a mean TLG of 2742.2.

The population characteristics are summarized in Table 1.

**Table 1:** Population characteristics, in particular gender, previous therapies, lesion characteristics such as vascularization and necrosis, data collected from ceCT, [^18^F]-FDG-PET/CT, and laboratory data.

		***n***	**%**	**mOS**	**Univariate**				**Multivariate**			
					**HR**	**CI 95%**		***p***	**HR**	**CI 95%**		***p***
Patients		45	100.0									
Gender	F	20	44.4	13.7	*ref*							
	M	25	55.6	9.8	1.276	0.672	2.423	0.457				
Prior Therapies	No Therapy	14	31.1	12.7	*ref*							
	Chemotherapy	14	31.1	4.2	1.151	0.493	2.684	0.745				
Surgery	11	24.4	16.9	1.013	0.437	2.350	0.975				
Loco-regional	6	13.3	13.3	0.657	0.224	1.930	0.445				
Vascularization	Peripheral	43	95.6									
	Ipodense	1	2.2									
	Hyperdense	1	2.2									
Necrosis	Absent	10	22.2	18.1	*ref*							
	Present	35	77.8	10.9	1.986	0.900	4.382	0.890				
CECT	T1	5	11.1	27.2	*ref*							
	T2	19	42.2	12.7	2.683	0.767	9.381	0.122				
	**T3**	21	46.7	7.9	3.725	1.077	12.889	**0.038**				
	N0	24	53.3	13.7	*ref*							
	N1	21	46.7	9.8	1.443	0.757	2.749	0.265				
	M0	20	44.4	13.7	*ref*							
	M1	25	55.6	12.3	1.726	0.891	3.343	0.106				
[^18^F]-FDG-PET/CT	T0	2	4.4	17.1	0.883	0.110	7.084	0.907				
	T1	13	28.9	15.7	*ref*							
	T2	19	42.2	13.3	1.453	0.644	3.278	0.368				
	T3	11	24.4	4.8	2.419	0.991	5.908	0.052				
	N0	41	91.1	13.3	*ref*							
	**N1**	4	8.9	1.6	1.048	0.304	3.611	0.941	6.261	0.752	52.144	**0.090**
	M0	24	53.3	16.7	*ref*							
	**M1**	21	46.7	4.9	1.982	1.041	3.776	**0.037**				
		**Mean**		**SD**								
Age (y)		62.9	±	13.2	0.993	1.041	3.776	0.506				
CECT	**max Ø (mm)**	76.2	±	38.1	1.013	1.004	1.023	**0.007**				
	**>** **Lesion Vol (cm**^**3**^**)**	321.7	±	408.6	2.023	1.138	3.595	**0.016**				
	**Tot Lesion Vol (cm** ^**3**^ **)**	414.9	±	577.2	2.261	1.225	4.173	**0.009**				
[^18^F]-FDG-PET/CT	**SUVmax**	12.8	±	8.2	1.050	1.012	1.091	**0.010**	1.091	1.040	1.114	**<0.001**
	**MTV**	479.0	±	667.1	4.256	2.132	8.497	**<0.001**				
	**TLG**	2,742.2	±	4,179.7	3.433	1.900	6.205	**<0.001**				
Lab Data	AST (U/L)	57.7	±	44.9	1.610	0.382	6.783	0.516				
	ALT (U/L)	53.7	±	64.0	0.972	0.323	2.921	0.960				
	**GGT (U/L)**	360.7	±	420.1	2.621	1.217	5.645	**0.014**	2.634	1.094	6.344	**0.031**
	**ALP (U/L)**	253.8	±	187.8	4.679	1.290	16.967	**0.019**				
	Tot Bilirubin (mg/dL)	1.5	±	2.0	2.170	0.652	7.225	0.207				
	**CEA (ng/mL)**	8.8	±	22.2	2.781	1.391	5.557	**0.004**				
	**CA 19-9 (U/mL)**	815.2	±	2,697.0	2.923	1.717	4.979	**<0.001**	2.043	1.173	3.558	**0.012**
	AFP (ng/mL)	40.7	±	126.2	1.000	0.995	1.004	0.811				

Mean OS follow-up: 47.9 months. n, number of patients; %, percentage of patients; mOS, months of overall survival; HR, hazard ratio; CI95%, confidence interval 95% p, probability index; N1, positive lymphnode; M1, positive metastasis; max Ø (mm), maximum diameter in mm; Mean, mean value; SD, standard deviation; > lesion Vol (cm3), volume in cm3 of the biggest lesion; Tot lesion vol (cm3), total volume of all the lesions in cm3; SUV msx maximum, standardized uptake value; MTV, metabolic tumor volume; TLG, total lesion glicolysis; GGT, Gamma-glutamyl Transferase; ALP, Alkaline Phosphatase; CEA, carcinoembryonic antigen; CA 19-9, Carbohydrate antigen 19-9. In bold are the values with the lowest Pearson correlation coefficient, with the corresponding name also bolded.

### 3.2. Determinants of survival

According to the univariate analysis, the parameters correlated with OS in a statistically significant way were as follows: at ceCT, T3 stage (HR = 3.725; *p* = 0.038), maximum transaxial lesion diameter (HR = 1.013; *p* = 0.007), major lesion volume (HR = 2.023; *p* = 0.016) and total volume disease (HR = 2.261; *p* = 0.009); at [18F]-FDG-PET/CT, presence of distant metastases (HR = 1.982; *p* = 0.037), SUVmax (HR = 1.050; *p* = 0.010), MTV (HR = 4.256; *p* < 0.001), and TLG (HR = 3. 433; *p* < 0.001); at biochemical analysis, GGT (HR = 2.621; *p* = 0.014), ALP (HR = 4.679; *p* = 0.019), CEA (HR = 2.781; *p* = 0.004), and CA 19-9 (HR = 2.923; *p* < 0.001).

Moreover, the following parameters did not correlate with OS in a statistically significant manner: at ceCT, stage ≤ T2, stage N and M; at [18F]-FDG-PET/CT, stage T and N; gender, prior therapies, presence of intralesional necrosis, AST, ALT, total bilirubin, and AFP.

**Figure 2:**
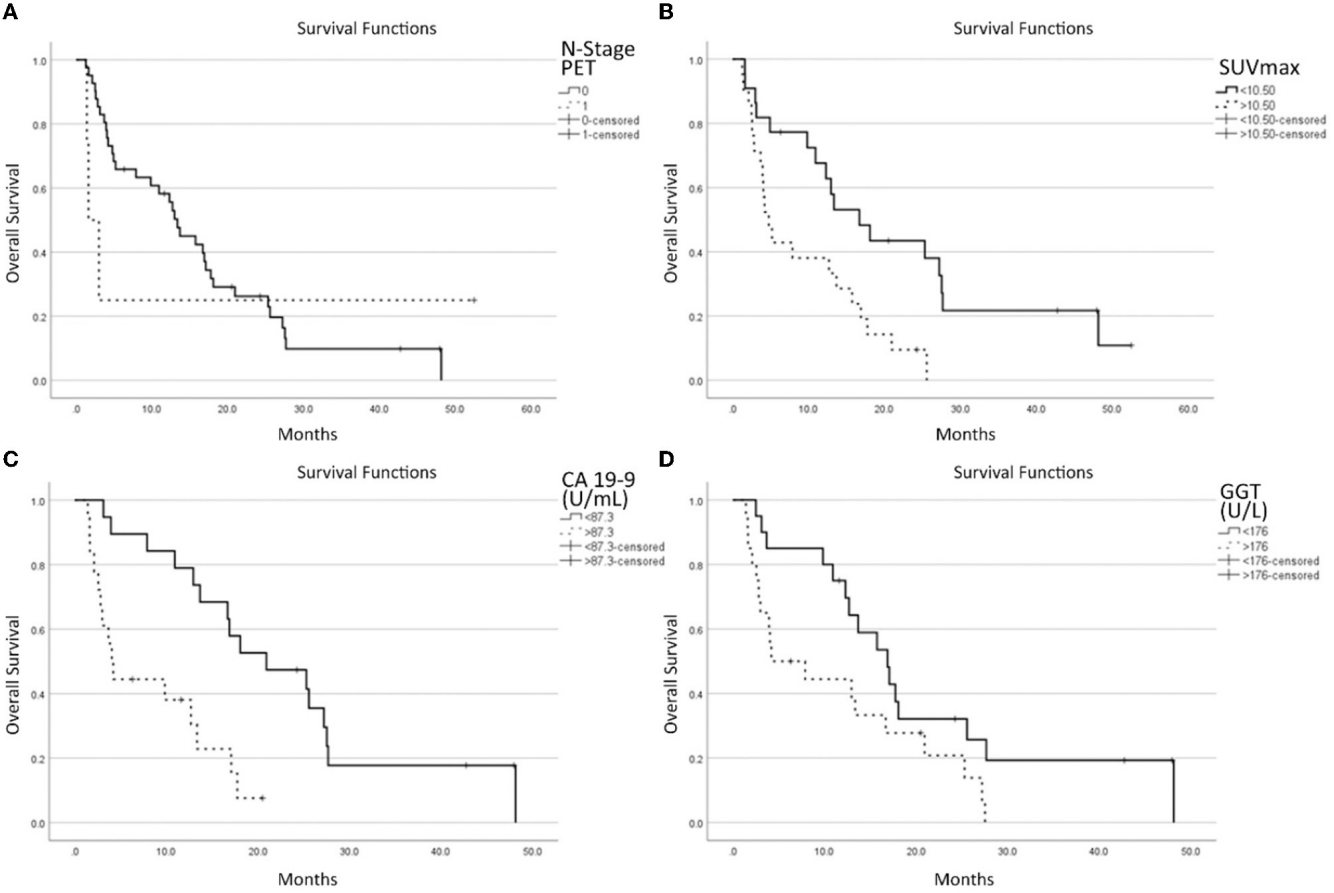
Survival functions in relation to the N stage at [^18^F]-FDG-PET/CT **(A)**, SUVmax **(B)**, CA 19-9 **(C)** GGT, and **(D)** laboratory data.

On multivariate analysis, the presence of lymph node metastases at PET scan (HR = 6.261; *p* = 0.090), SUVmax (HR = 1.091; *p* < 0.001), GGT (HR = 2.634; *p* = 0.031), and CA 19-9 (HR = 2.043; *p* = 0.012) were found to be independent prognostic factors for OS (Figure 2).

Regarding the survival curve of N1 patients, it must be noted that it is much steeper than that for the N0 stage in the first 5–10 months, and then it is flat. This is because the clinical conditions of three patients worsened rapidly while one is still alive after more than 4 years. One patient behaved totally differently from the other 3. This heavily impacts on the statistical analysis and explains why the *p*-value (*p* = 0.09) is the highest among the four parameters selected by the multivariate analysis, next to the limit of acceptability.

Dividing the patient population according to the pre-enrolling care, these parameters did not reach a statistically significant level (*P* = 0.149). Furthermore, pre-enrollment care appears to be unrelated to N-positive/negative lymph nodes (*P* = 1.000) nor superior/inferior SUVmax of lesions (*P* = 0.132).

**Figure 3:**
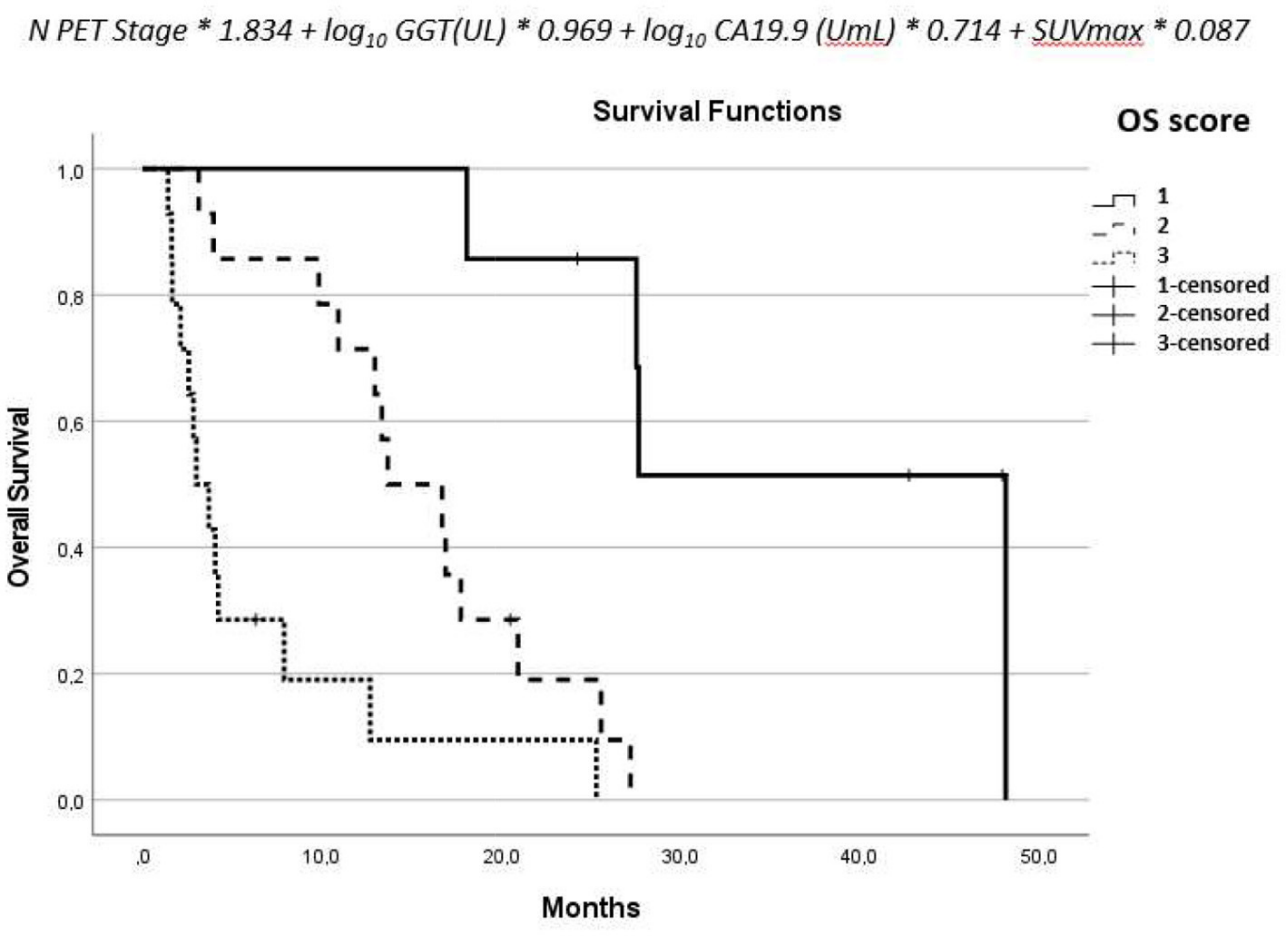
Formula derived from the multivariate analysis and the overall survival of the three groups.

The aforementioned derived score of OS combines PET, CT, and clinical data. It showed to be correlated to OS in a statistically significant way: for patients with a score of 1, mOS = 48.1; for a score of 2, mOS = 13.7 (HR: 15.9; *p* = 0.009); for a score of 3, mOS = 3.0 (HR: 53.8; *p* < 0.001) (Figure 3).

The results derived from the score were confirmed after splitting the population in treated patients (overall log-rank test for OS: *P* < 0.001), and naive patients (overall log-rank test for OS: *P* = 0.016).

## 4. Discussion

According to the latest National Comprehensive Cancer Network (NCCN) guidelines, the imaging evaluation of iCCA should include hepatic lesion extension, lymph node, and distant metastases, considering that metastatic lymph nodes over the hepatic hilum and hepatic metastases should be considered as distant metastases, preventing from curative surgery (27).

The ceCT offers a high prognostic value in terms of survival and the vascularization and the diameter embody the main biomarkers for this assessment. Actually, it is widely well-known in the scientific literature that enhancement patterns correlate with tumor spread and survival (28–30). The ICC patterns are hypovascular, rim-like, and hypervascular. The first one shows the highest frequency of lymphatic, perineural, and biliary dissemination, and a low survival rate at 1 and 3 years, with 30.7 and 0%, respectively. On the contrary, the hypervascular ICC pattern displays lower rates of invasion and the longest survival at 1 and 3 years, with 88.9 and 66.7%, respectively (27). However, in our study, the largest part of patients presented a rim-like pattern, and consequently, this parameter did not affect the prognostic score proposed. Moreover, the diameter depicts the second key factor that should be taken into account for a prognostic evaluation of ceCT in patients affected by ICC. As shown in literature, the diameter represents a crucial factor in radiomics studies which allows to predict both patients with low risk of early intrahepatic recurrence and the response to target therapies, such as TARE (31, 32). Indeed, in accordance with the literature, in our study, the diameter is confirmed as a parameter statistically significant according to the univariate analysis and therefore correlated with survival.

Regarding [18F]-FDG-PET/CT, many studies in the literature have already highlighted its crucial role in the oncological management of patients with cholangiocarcinoma at the diagnostic stage (11, 33–35), as well as a statistical correlation between semi-quantitative parameters such as SUVmax, MTV, and TLG with OS (21, 22).

In our study, we have demonstrated that the combination of FDG PET/CT, multiphase ceCT, and laboratory data retain a prognostic value even in patients who are not candidates for surgery regardless of the subsequent therapeutical option (TARE vs. systemic treatment).

Despite the observation that an important discordance between the two imaging methods in the assessment of lymph node involvement with [18F]-FDG-PET/CT showing a lower detection rate as compared to ceCT, an important correlation of PET N+ results with OS was found. This is probably due to the fact that the disease metabolic rate detected through the FDG uptake significantly impacts prognosis, while metastatic lymph nodes with a low metabolic rate (and consequently no FDG uptake) retain a lower prognostic meaning.

The main limitations of our study were the relatively low number of enrolled patients and the heterogeneity of pre-enrollment care. As patients with iCCA potentially candidate to TARE is not a common clinical condition, the therapeutical approach is not standardized yet (36). In our experience, [18F]-FDG-PET/CT has proven to be crucial both for correctly assessing the extent of intra and extrahepatic disease significantly impacting subsequent treatment choice when associated with multiphase ceCT. This is witnessed by the relatively high number of patients who came to our center for TARE and were not treated after the imaging evaluation. This suggests that the routine use of [18F]-FDG-PET/CT and ceCT in clinical practice may have a significant impact on the therapeutic management of patients with iCCA and could potentially be proposed as a new imaging standard for the evaluation of TARE candidate patients.

Considering that, in addition to the parameters derived from multimodality imaging, laboratory data also showed a significant correlation with patient prognosis: we believe that the nomogram that we elaborated, combining [18F]-FDG-PET/CT, ceCT, and laboratory data, can be considered a useful tool for prognostic stratification.

## 5. Conclusion

Considering our data, performing integrated multimodality imaging is critical for the prognostic stratification of patients with iCCA.

Indeed, in our experience, while ceCT remains the reference imaging method in the staging of local disease, [18F]-FDG-PET/CT has proven to be crucial in the lymph node and distant disease evaluation. In addition, semi-quantitative parameters, such as SUVmax, MTV, and TLG were also effective in the prognostic stratification of patients.

## Data availability statement

The raw data supporting the conclusions of this article will be made available by the authors, without undue reservation.

## Ethics statement

The studies involving human participants were reviewed and approved by Review Board and Ethical Committee of S. Orsola Hospital (193/2021/Oss/AOUBo). The patients/participants provided their written informed consent to participate in this study.

## Author contributions

CN and CM: conceptualization and investigation. APac: methodology and formal analysis. VD and MB: data curation. CN, CM, and MC: manuscript writing. SB and NF, and APal: manuscript writing-review and editing. GB and SF: supervision. All authors have read and agreed to the published version of the manuscript.

## References

[1] Welzel TM McGlynn KA Hsing AW O'Brien TR Pfeiffer RM. Impact of classification of hilar cholangiocarcinomas (Klatskin tumors) on the incidence of intra- and extrahepatic cholangiocarcinoma in the United States. J Natl Cancer Inst. (2006) 98:873–5. 10.1093/jnci/djj234 16788161

[2] Rizvi S Gores GJ. Pathogenesis, diagnosis, and management of cholangiocarcinoma. Gastroenterology. (2013) 145:1215–29. 10.1053/j.gastro.2013.10.013 24140396 PMC3862291

[3] Bergquist A von Seth E. Epidemiology of cholangiocarcinoma. Best Pract Res Clin Gastroenterol. (2015) 29:221–32. 10.1016/j.bpg.2015.02.003 25966423

[4] Blechacz B Komuta M Roskams T Gores GJ. Clinical diagnosis and staging of cholangiocarcinoma. Nat Rev Gastroenterol Hepatol. (2011) 8:512–22. 10.1038/nrgastro.2011.131 21808282 PMC3331791

[5] Shaib Y El-Serag HB. The epidemiology of cholangiocarcinoma. Semin Liver Dis. (2004) 24:115–25. 10.1055/s-2004-828889 15192785

[6] Shaib YH Davila JA McGlynn K El-Serag HB. Rising incidence of intrahepatic cholangiocarcinoma in the United States: a true increase? J Hepatol. (2004) 40:472–7. 10.1016/j.jhep.2003.11.030 15123362

[7] Khan SA Toledano MB Taylor-Robinson SD. Epidemiology, risk factors, and pathogenesis of cholangiocarcinoma. HPB. (2008) 10:77–82. 10.1080/13651820801992641 18773060 PMC2504381

[8] Blechacz BR Gores GJ. Cholangiocarcinoma. Clin Liver Dis. (2008) 12:131–50. 10.1016/j.cld.2007.11.003 18242501

[9] Weber SM Ribero D O'Reilly EM Kokudo N Miyazaki M Pawlik TM. Intrahepatic cholangiocarcinoma: expert consensus statement. HPB. (2015) 17:669–80. 10.1111/hpb.12441 26172134 PMC4527852

[10] Spolverato G Vitale A Cucchetti A Popescu I Marques HP Aldrighetti L. Can hepatic resection provide a long-term cure for patients with intrahepatic cholangiocarcinoma? Cancer. (2015) 121:3998–4006. 10.1002/cncr.29619 26264223

[11] Petrowsky H Wildbrett P Husarik DB Hany TF Tam S Jochum W. Impact of integrated positron emission tomography and computed tomography on staging and management of gallbladder cancer and cholangiocarcinoma. J Hepatol. (2006) 45:43–50. 10.1016/j.jhep.2006.03.009 16690156

[12] Tsunematsu S Chuma M Kamiyama T Miyamoto N Yabusaki S Hatanaka K. Intratumoral artery on contrast-enhanced computed tomography imaging: differentiating intrahepatic cholangiocarcinoma from poorly differentiated hepatocellular carcinoma. Abdom Imaging. (2015) 40:1492–9. 10.1007/s00261-015-0352-9 25579172

[13] Zhang Y Uchida M Abe T Nishimura H Hayabuchi N Nakashima Y. Intrahepatic peripheral cholangiocarcinoma: comparison of dynamic CT and dynamic MRI. J Comput Assist Tomogr. (1999) 23:670–7. 10.1097/00004728-199909000-00004 10524843

[14] Vilgrain V. Staging cholangiocarcinoma by imaging studies. HPB. (2008) 10:106–9. 10.1080/13651820801992617 18773065 PMC2504386

[15] Okami J Dono K Sakon M Tsujie M Hayashi N Fujiwara Y. Patterns of regional lymph node involvement in intrahepatic cholangiocarcinoma of the left lobe. J Gastrointest Surg. (2003) 7:850–6. 10.1007/s11605-003-0029-5 14592657

[16] Baheti AD Tirumani SH Shinagare AB Rosenthal MH Hornick JL Ramaiya NH. Correlation of CT patterns of primary intrahepatic cholangiocarcinoma at the time of presentation with the metastatic spread and clinical outcomes: retrospective study of 92 patients. Abdom Imaging. (2014) 39:1193–201. 10.1007/s00261-014-0167-0 24869789

[17] You MW Yun SJ. Differentiating between hepatocellular carcinoma and intrahepatic cholangiocarcinoma using contrast-enhanced MRI features: a systematic review and meta-analysis. Clin Radiol. (2019) 74:406. 10.1016/j.crad.2018.12.016 30704667

[18] Annunziata S Caldarella C Pizzuto DA Galiandro F Sadeghi R Giovanella L. Diagnostic accuracy of fluorine-18-fluorodeoxyglucose positron emission tomography in the evaluation of the primary tumor in patients with cholangiocarcinoma: a meta-analysis. Biomed Res Int. (2014) 2014:247693. 10.1155/2014/247693 24955351 PMC4052790

[19] Corvera CU Blumgart LH Akhurst T DeMatteo RP D'Angelica M Fong Y. 18F-fluorodeoxyglucose positron emission tomography influences management decisions in patients with biliary cancer. J Am Coll Surg. (2008) 206:57–65. 10.1016/j.jamcollsurg.2007.07.002 18155569

[20] Moon CM Bang S Chung JB Park SW Song SY Yun M. Usefulness of 18F-fluorodeoxyglucose positron emission tomography in differential diagnosis and staging of cholangiocarcinomas. J Gastroenterol Hepatol. (2008) 23:759–65. 10.1111/j.1440-1746.2007.05173.x 17931372

[21] Ma KW Cheung TT She WH Chok KSH Chan ACY Dai WC Chiu WH. Diagnostic and prognostic role of 18-FDG PET/CT in the management of resectable biliary tract cancer. World J Surg. (2018) 42:823–34. 10.1007/s00268-017-4192-3 28905105

[22] Ikeno Y Seo S Iwaisako K Yoh T Nakamoto Y Fuji H. Preoperative metabolic tumor volume of intrahepatic cholangiocarcinoma measured by 18F-FDG-PET is associated with the KRAS mutation status and prognosis. J Transl Med. (2018) 16:95. 10.1186/s12967-018-1475-x 29642912 PMC5896043

[23] Boellaard R Delgado-Bolton R Oyen WJ Giammarile F Tatsch K Eschner W. FDG PET/CT: EANM procedure guidelines for tumour imaging: version 20. Eur J Nucl Med Mol Imaging. (2015) 42:328–54. 10.1007/s00259-014-2961-x 25452219 PMC4315529

[24] Iatrou M Ross SG Manjeshwar RM Stearns CW. A fully 3D iterative image reconstruction algorithm incorporating data corrections. IEEE Symposium Conf. Rec. Nucl. Sci. (2004) 4:2493–7. 10.1109/NSSMIC.2004.1462761

[25] Mosconi C Gramenzi A Ascanio S Cappelli A Renzulli M Pettinato C. Yttrium-90 radioembolization for unresectable/recurrent intrahepatic cholangiocarcinoma: a survival, efficacy and safety study. Br J Cancer. (2016) 115:297–302. 10.1038/bjc.2016.191 27336601 PMC4973156

[26] Gramenzi A Golfieri R Mosconi C Cappelli A Granito A Cucchetti A. Yttrium-90 radioembolization vs sorafenib for intermediate-locally advanced hepatocellular carcinoma: a cohort study with propensity score analysis. Liver Int. (2015) 35:1036–47. 10.1111/liv.12574 24750853

[27] Fujita N Asayama Y Nishie A Ishigami K Ushijima Y Takayama Y. Mass-forming intrahepatic cholangiocarcinoma: enhancement patterns in the arterial phase of dynamic hepatic CT - correlation with clinicopathological findings. Eur Radiol. (2017) 27:498–506. 10.1007/s00330-016-4386-3 27165138

[28] Hewitt DB Brown ZJ Pawlik TM. Surgical management of intrahepatic cholangiocarcinoma. Expert Rev Anticancer Ther. (2022) 22:27–38. 10.1080/14737140.2022.1999809 34730474

[29] Panettieri E Maki H Kim BJ Kang HC Cox V Vega EA. Arterial enhancement pattern predicts survival in patients with resectable and unresectable intrahepatic cholangiocarcinoma. Surg Oncol. (2022) 40:101696. 10.1016/j.suronc.2021.101696 34995974 PMC8863406

[30] Park HM Jang HY Lee DE Kang MJ Han SS. Prognostic impact of tumor vascularity on CT in resectable intrahepatic cholangiocarcinoma. HPB. (2022) 24:359–69. 10.1016/j.hpb.2021.06.424 34325966

[31] Jolissaint JS Wang T Soares KC Chou JF Gönen M Pak LM. Machine learning radiomics can predict early liver recurrence after resection of intrahepatic cholangiocarcinoma. HPB. (2022) 24:1341–50. 10.1016/j.hpb.2022.02.004 35283010 PMC9355916

[32] Mosconi C Cucchetti A Bruno A Cappelli A Bargellini I De Benedittis C. Radiomics of cholangiocarcinoma on pretreatment CT can identify patients who would best respond to radioembolisation. Eur Radiol. (2020) 30:4534–44. 10.1007/s00330-020-06795-9 32227266

[33] Kluge R Schmidt F Caca K Barthel H Hesse S Georgi P. Positron emission tomography with [(18)F]fluoro-2-deoxy-D-glucose for diagnosis and staging of bile duct cancer. Hepatology. (2001) 33:1029–35. 10.1053/jhep.2001.23912 11343227

[34] Kato T Tsukamoto E Kuge Y Katoh C Nambu T Nobuta A. Clinical role of (18)F-FDG PET for initial staging of patients with extrahepatic bile duct cancer. Eur J Nucl Med Mol Imaging. (2002) 29:1047–54. 10.1007/s00259-002-0852-z 12173019

[35] Fritscher-Ravens A Bohuslavizki KH Broering DC Jenicke L Schäfer H Buchert R. in the diagnosis of hilar cholangiocarcinoma. Nucl Med Commun. (2001) 22:1277–85. 10.1097/00006231-200112000-00002 11711897

[36] Valle JW Borbath I Khan SA Huguet F Gruenberger T Arnold D. ESMO Guidelines Committee. Biliary cancer: ESMO clinical practice guidelines for diagnosis, treatment and follow-up. Ann Oncol. (2016) 27:v28–37. 10.1093/annonc/mdw324 27664259

